# Supplementary material for: Research Progress on the Effects of Selenium on the Growth and Quality of Tea Plants
Source: Plants (Basel). 2022 Sep 23;11(19):2491. doi: 10.3390/plants11192491 (PMC9573726; doi:10.3390/plants11192491)
Supplement: Supplementary file 1 [file plants-1893907-supplementary.pdf › plants-1893907-supplementary.pdf]

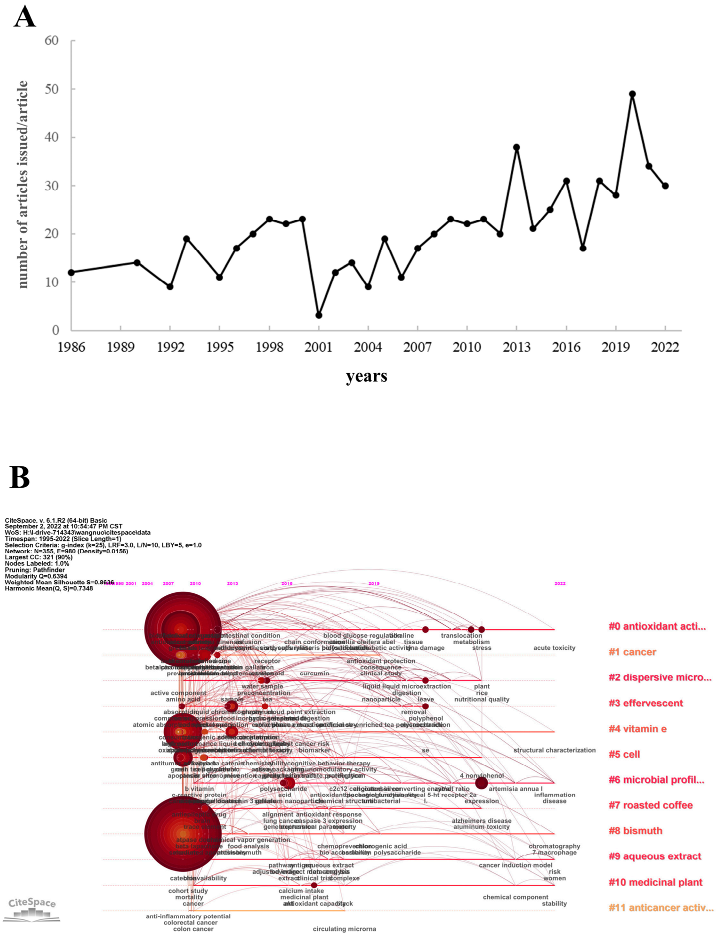

Figure S1. (**A**), Annual distribution of literatures quantity; (**B**), keyword co-occurrence time map in Web of Science™ database.
